# Signal Transduction Regulators in Axonal Regeneration

**DOI:** 10.3390/cells11091537

**Published:** 2022-05-04

**Authors:** Barbara Hausott, Rudolf Glueckert, Anneliese Schrott-Fischer, Lars Klimaschewski

**Affiliations:** 1 Institute of Neuroanatomy, Medical University Innsbruck, 6020 Innsbruck, Austria; lars.klimaschewski@i-med.ac.at; 2 Department of Otorhinolaryngology, Medical University Innsbruck, 6020 Innsbruck, Austria; rudolf.glueckert@i-med.ac.at (R.G.); annelies.schrott@i-med.ac.at (A.S.-F.)

**Keywords:** Sprouty, PTEN, ERK, Akt, ubiquitin ligase, microRNA, axon regeneration

## Abstract

Intracellular signal transduction in response to growth factor receptor activation is a fundamental process during the regeneration of the nervous system. In this context, intracellular inhibitors of neuronal growth factor signaling have become of great interest in the recent years. Among them are the prominent signal transduction regulators Sprouty (SPRY) and phosphatase and tensin homolog deleted on chromosome 10 (PTEN), which interfere with major signaling pathways such as extracellular signal-regulated kinase (ERK) or phosphoinositide 3-kinase (PI3K)/Akt in neurons and glial cells. Furthermore, SPRY and PTEN are themselves tightly regulated by ubiquitin ligases such as c-casitas b-lineage lymphoma (c-CBL) or neural precursor cell expressed developmentally down-regulated protein 4 (NEDD4) and by different microRNAs (miRs) including miR-21 and miR-222. SPRY, PTEN and their intracellular regulators play an important role in the developing and the lesioned adult central and peripheral nervous system. This review will focus on the effects of SPRY and PTEN as well as their regulators in various experimental models of axonal regeneration in vitro and in vivo. Targeting these signal transduction regulators in the nervous system holds great promise for the treatment of neurological injuries in the future.

## 1. Introduction

**Figure 1:**
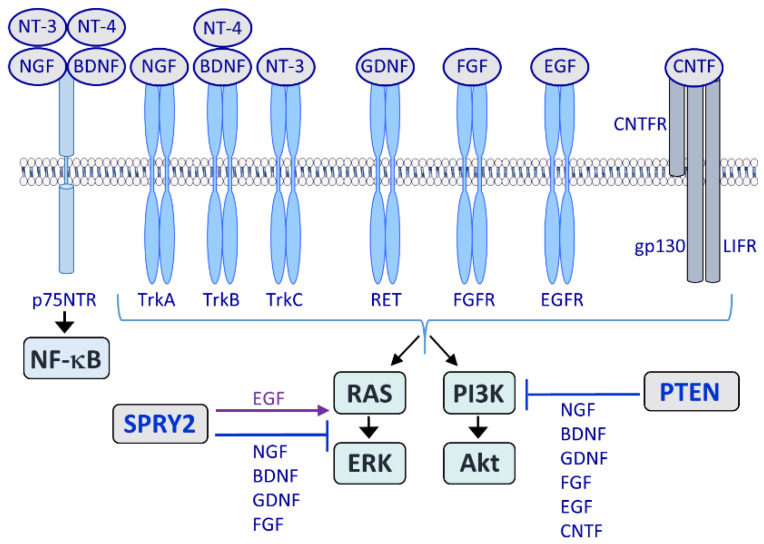
Major growth factor receptors acting in the NS are the tropomyosin receptor kinases (Trks) TrkA, -B and -C, which are activated by nerve growth factor (NGF), brain-derived neurotrophic factor (BDNF), neurotrophin 3 (NT-3) and NT-4. The p75 neurotrophin receptor (p75NTR) acts as a co-receptor for Trks and induces nuclear factor-kappa B (NF-κB). Glial cell-line-derived neurotrophic factor (GDNF) activates the ‘rearranged during transfection’ (RET) receptor tyrosine kinase (RTK) and among the 22 fibroblast growth factors (FGFs) and four types of FGF receptors (FGFRs), FGF1 and FGF2 together with FGFR1 play a major role during nervous system (NS) regeneration. By contrast, epidermal growth factor receptor (EGFR) activation by epidermal growth factor (EGF) limits regeneration. The neuropoietic cytokine ciliary neurotrophic factor (CNTF) belongs to the gp130 receptor family and the CNTF receptor (CNTFR) is composed of three chains: A specific CNTFR chain, gp130 and leukemia inhibitory factor receptor (LIFR). LIFR does not have intrinsic tyrosine kinase activity but the gp130 and LIFRβ chains are constitutively associated with members of the Janus kinase (JAK) family of tyrosine kinases. The rat sarcoma (RAS)/extracellular signal-regulated kinase (ERK) and the phosphoinositide 3-kinase (PI3K)/Akt pathways are activated by all major growth factor and cytokine families. Sprouty2 (SPRY2) inhibits RAS/ERK signaling in response to NGF, BDNF, GDNF and FGF whereas EGF signaling is enhanced by SPRY proteins. Phosphatase and tensin homolog deleted on chromosome 10 (PTEN) inhibits PI3K/Akt signaling in response to NGF, BDNF, GDNF, FGF, EGF and CNTF.

The peripheral nervous system (PNS) is provided with the intrinsic capability to regenerate following injury, although the regeneration rate is slow and functional outcomes are often poor [1,2,3]. In contrast, the regeneration of the central nervous system (CNS) is still highly limited due to the inhibitory effects of molecules associated with CNS myelin and a weak intrinsic capacity for axon growth [4,5,6]. Intracellular signal transduction in response to growth factor receptor activation is a fundamental process during nervous system (NS) regeneration in the PNS and the CNS. The activation of the prominent signal cascades rat sarcoma (RAS)/extracellular signal-regulated kinase (ERK) and phosphoinositide 3-kinase (PI3K)/Akt promotes regeneration processes [1,7,8]. These intracellular signaling molecules are activated by growth factors binding to receptor tyrosine kinases (RTKs) on the cell surface (Figure 1). Major growth factor receptors in the NS are the neurotrophin receptor tropomyosin receptor kinases (Trks) TrkA, -B and -C, which are activated by nerve growth factor (NGF), brain-derived neurotrophic factor (BDNF), neurotrophin 3 (NT-3) and NT-4, respectively. All neurotrophins also interact with the p75 neurotrophin receptor (p75NTR) which belongs to the tumor necrosis factor receptor family. The p75NTR may enhance or suppress Trk activity and it induces apoptosis or survival autonomously through Jun N-terminal kinase (JNK) and nuclear factor-kappa B (NF-κB) [9,10]. NF-κB stimulates or inhibits axon growth of cultured developing neurons depending on the type of neuron and the neurotrophic factor involved [11]. In the adult brain NF-κB initiates the renewal of neurons and induces axogenesis of newborn granule cells [12]. NF-κB is also involved in glial scar formation after injury and astroglial inhibition of NF-κB facilitates regeneration [13,14]. The glial cell-line-derived neurotrophic factor (GDNF) family members include GDNF, neurturin (NRTN), persephin (PSPN) and artemin (ARTN) which activate the ‘rearranged during transfection’ (RET) RTK. Neuropoietic cytokines belonging to the gp130 receptor family such as ciliary neurotrophic factor (CNTF), leukemia inhibitory factor (LIF) or interleukins (IL) are also involved in NS regeneration among others [15]. Moreover, other growth factors that are not specific for the NS, such as fibroblast growth factors (FGFs), have crucial functions during nerve regeneration. Among the 22 members of the FGF family and the four types of FGF receptors (FGFRs), FGF1 and FGF2 (acidic and basic FGF) as well as FGFR1 play a major role during NS regeneration [16]. Epidermal growth factor (EGF) signaling is important during NS development, but it inhibits the regeneration of the CNS. EGF receptor (EGFR) inhibitors limit the release of inhibitory molecules after injury and stimulate the production of neurotrophic factors [17].

The induction of the RAS/ERK and the PI3K/Akt pathway by different growth factors and cytokines overlaps but the duration and strength of their activation differs. EGF induces transient ERK activation which leads to the proliferation of PC12 pheochromocytoma cells, whereas FGF2 and NGF result in sustained ERK activation that is required for neurite outgrowth [18,19]. Activation of ERK by different FGFs is commonly observed whereas the activation of Akt differs among FGF isoforms and cell types [20]. In dorsal root ganglion (DRG) neurons, the activation of ERK is induced by FGF2 and NGF to a similar extent whereas Akt is much stronger activated by NGF than by FGF2 [21]. In comparison, NT-3 is a stronger activator of Akt than NGF in PC12 cells and DRG cultures [22]. In superior cervical ganglion (SCG) neuron cultures, both NGF and BDNF induce the activation of ERK and Akt to a similar extent [23,24]. GDNF induces sustained phosphorylation of Akt and ERK in neuroectodermic cells [25]. CNTF enhances short-term ERK phosphorylation whereas PI3K/Akt activation is delayed although it is crucial for motoneuron survival [26].

**Figure 2:**
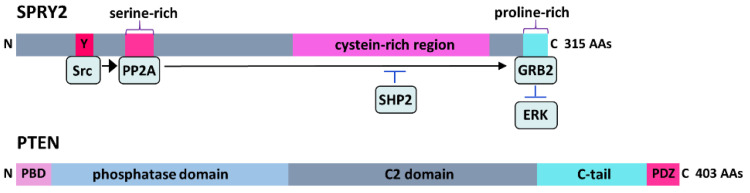
SPRY2 is a 315-amino acid (AA) protein containing a variable N-terminal region with various phosphorylation sites and a highly conserved C-terminal cystein-rich region. RTK stimulation induces tyrosine (Y) phosphorylation in the N-terminus of SPRY2 by Src kinase, which serine dephosphorylates SPRY2 by protein phosphatase 2A (PP2A). This results in a conformational change at the C-terminal proline-rich binding site for growth factor receptor-bound protein 2 (GRB2), thereby preventing the interaction of GRB2 with son of sevenless (SOS) and subsequent ERK activation, while its dephosphorylation by Src homology 2-containing phosphotyrosine phosphatase (SHP2) dissociates it from GRB2. PTEN is a 403-AA enzyme containing the N-terminal phosphatidylinositol 4,5-bisphosphate (PIP2)-binding domain (PBD), a phosphatase domain, a membrane-targeting C2 domain, a C-terminal tail and a PDZ-binding motif that regulates its stability and subcellular localization.

Intracellular inhibitors of neuronal growth factor signaling have become of great interest in the recent years in neuroscience. The signal transduction modulators Sprouty (SPRY; SPRY2 https://www.uniprot.org/uniprot/O43597; accessed on 1 April 2022) and phosphatase and tensin homolog deleted on chromosome 10 (PTEN; https://www.uniprot.org/uniprot/P60484; accessed on 1 April 2022) [27] are present in the PNS and the CNS (Figure 2). Furthermore, microRNAs (miRs) and ubiquitin ligases are involved in the regulation of SPRY and PTEN, among others. This review focuses on the signal transduction regulators SPRY and PTEN together with related miRs and ubiquitin ligases involved in axon growth during the development and regeneration of the NS. The fact that SPRY and PTEN play a major role in the regulation of neuronal survival and axonal specification during development, supports their function during axon regeneration in the adult NS. 

## 2. The Role of RAS/ERK and PI3K/Akt Signaling in Axonal Regeneration

**Figure 3:**
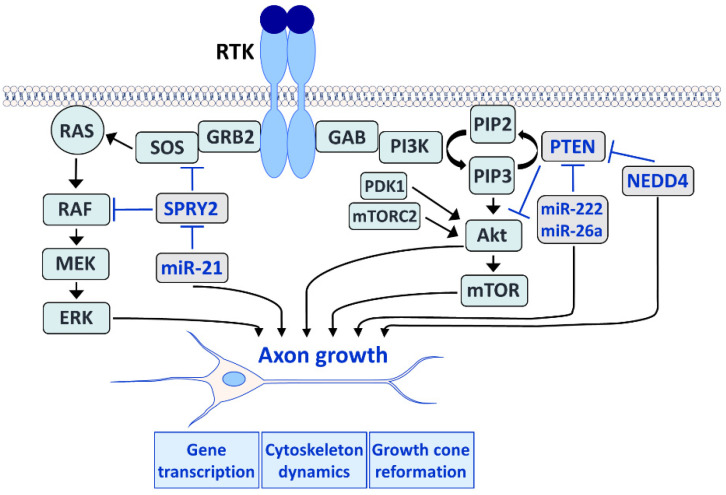
The RAS/ERK (left) and the PI3K/Akt (right) pathways play an important role in axon growth during development and regeneration in the adult. Both pathways induce gene expression in the neuronal cell body and control cytoskeleton dynamics in the axon. Furthermore, both pathways influence the growth cone reformation after lesion. The RAS/ERK pathway (left) is induced after RTK activation by the GRB2 adaptor molecule that recruits SOS, which in turn activates RAS. RAS recruits rapidly accelerated fibrosarcoma (RAF) to the plasma membrane where it is activated and induces mitogen-activated and extracellular signal-regulated kinase (MEK) that phosphorylates ERK. SPRY2 interacts with the ERK pathway by interfering with GRB2 and RAF. Activation of ERK is increased by SPRY2 deletion and enhances axon regeneration. MicroRNA-21 (miR-21) downregulates SPRY2 and is induced after nerve injury to promote axon regeneration. The PI3K/Akt pathway (right) is induced by the adaptor protein GRB2-associated binder (GAB) which activates PI3K. Activated PI3K phosphorylates PIP2 to generate phosphatidylinositol 3,4,5-trisphosphate (PIP3). The accumulation of PIP3 recruits Akt to the plasma membrane, and Akt is phosphorylated by phosphoinositide-dependent kinase 1 (PDK1) and mammalian target of rapamycin (mTOR) complex 2 (mTORC2). Akt then activates mTOR, and both Akt and mTOR promote axon regeneration. PTEN converts PIP3 to PIP2 by dephosphorylation, thereby reversing the reaction catalyzed by PI3K and inhibiting Akt and mTOR activation. MiR-222 and miR-26a downregulate PTEN and are regulated after nerve lesion or during brain development, respectively. Deletion of PTEN enhances axon regeneration via the activation of Akt and mTOR. MiR-222 and miR-26a promote axon growth by the downregulation of PTEN whereas the disruption of the PTEN-regulating ubiquitin ligase neural precursor cell expressed developmentally down-regulated protein 4 (NEDD4) upregulates PTEN and impairs axon growth.

The main function of RTK signaling is to provide the proteins required for survival and axon growth via changes in gene transcription and protein synthesis. Injured neurons upregulate regeneration-associated genes (RAGs) including those that express cytoskeletal components to form new axons and growth cones. Signaling of RAS/ERK and PI3K/Akt (Figure 3) is fundamental for the induction of RAGs during nerve regeneration [1,7,8,15,28]. Activation of RTKs results in dimerization and phosphorylation of tyrosine residues at the cytoplasmic site of the receptor. This creates binding sites for the growth factor receptor-bound protein 2 (GRB2) adaptor molecule that recruits the guanine nucleotide exchange factor son of sevenless (SOS) which activates RAS. RAS then recruits rapidly accelerated fibrosarcoma (RAF) to the membrane where it is activated. RAF further induces mitogen-activated and extracellular signal-regulated kinase (MEK) that phosphorylates ERK on both threonine and tyrosine residues in the cyto- and axoplasm of neurons [8,29].

Activation of ERK promotes axon elongation by cultured embryonic DRG and SCG neurons [22,23]. However, ERK inhibition has no effect on spontaneous axon outgrowth of adult DRG neurons while axon growth in response to NGF stimulation is impaired by ERK inhibition [30,31]. Furthermore, the activation of ERK is required for axotomy-induced growth cone reformation after lesion and for the polymerization of microtubules and actin filaments [23,24,32,33]. In addition, ERK is activated in the proximal and distal nerve stumps following sciatic nerve crush and inhibition of ERK impairs nerve regeneration in vivo [34,35]. Although ERK signaling is important for axon regeneration after injury, ERK is not a major mediator of neuronal survival after injury [35,36]. However, ERK plays a role in neuronal survival in response to toxicity [37,38].

Activated RTKs induce PI3K/Akt signaling through direct binding or through tyrosine phosphorylation of adaptor proteins such as GRB2-associated binder (GAB), which then activates PI3K. Activated PI3K phosphorylates the 3’ position of phosphatidylinositol 4,5-bisphosphate (PI4,5P2 or PIP2) to generate phosphatidylinositol 3,4,5-trisphosphate (PIP3). The accumulation of PIP3 recruits Akt to the plasma membrane, which binds to PIP3 via the pleckstrin homology (PH) domain. Akt is then phosphorylated at Thr308 and Ser473 by phosphoinositide-dependent kinase 1 (PDK1) and the mammalian target of rapamycin (mTOR) complex 2 (mTORC2), respectively, resulting in full activation [39]. There is evidence that in addition to the plasma membrane, an endomembrane pool of active Akt exists that is activated locally through PIP3 and PI3,4P2 [40]. Akt in turn inhibits glycogen synthase kinase 3 (GSK3) through the phosphorylation of its amino-terminal serine residue, which activates several transcription factors involved in axon growth and enhances cytoskeleton dynamics [41,42]. Furthermore, Akt activates mTOR via mTORC1 and induces phosphorylation of the ribosomal protein S6, which plays a major role in CNS regeneration [5]. 

Activation of Akt specifically increases the axon caliber and enhances axonal branching of embryonic DRG neurons [22]. In addition, Akt activation contributes to NGF-induced axonal branching of adult DRG neurons [43]. Activated Akt is present in cell bodies and growth cones of adult DRG neurons, and inhibition of Akt suppresses both spontaneous and growth factor-induced neurite outgrowth [44,45]. PI3K activity is also required for the redevelopment of adult DRG growth cones after laser injury, and PI3K inhibitors strongly inhibit growth cone formation even when applied exclusively to the axonal compartment through microfluidic separation [46]. In contrast, regenerative axon growth in response to a preconditioning lesion is not affected by PI3K inhibition in dissociated DRG cultures although phosphorylation of Akt is enhanced in regenerating nerves after sciatic nerve crush [7,47]. In the CNS, axonal PIP3 decreases at the time when neurons lose their regenerative ability. Overexpression of the p110δ isoform of PI3K enhances axonal PIP3 and promotes axon regeneration after optic nerve crush injury as well as survival of retinal ganglion cell (RGC) neurons [46]. This confirms that in addition to its role in axon growth, the PI3K pathway represents a major survival-promoting pathway in neurons [45,48,49]. 

Most growth factors activate both the RAS/ERK and the PI3K/Akt pathway. Studies that activate one signaling pathway independently are rare. Specific activation of optoRAF or optoAkt enhances axon regeneration in the PNS and the CNS of *Drosophila* larvae [50]. In embryonic DRG cultures, overexpression of RAF or Akt induces distinct axon morphologies, and co-expression of RAF and Akt results in additive effects on axon elongation and branching [22]. Although crosstalk between RAF and Akt was not observed in these studies, crosstalk between the two pathways has been reported in tumor cells [51]. During growth factor signaling, crosstalk between RAS/ERK and PI3K/Akt occurs upstream at the level of activated RAS which induces PI3K/Akt signaling [52]. Strong Akt activation inhibits ERK phosphorylation of adult DRG neurons indicating a crosstalk between these two pathways in neurons [53]. This is confirmed during brain ischemia when strong Akt phosphorylation suppresses ERK activation by phosphorylation of the inhibitory Ser259 of RAF [54,55].

## 3. SPRY: Signal Regulators of RAS/ERK Signaling

SPRY proteins were first described as antagonists of FGF signaling that control apical branching of the *Drosophila* airways. Inhibition of dSPRY induces excessive tracheal branching caused by enhanced FGF signaling [56]. In mammals, four SPRY genes (SPRY1-4) were identified with SPRY2 exhibiting the highest sequence homology to dSPRY, indicating its distinct evolutionary conservation [57,58,59,60]. SPRY proteins modulate RTK signaling in response to several growth factors including NGF [61,62], BDNF [63], GDNF [64] and FGF [56,61,65]. By contrast, EGF signaling is enhanced by SPRY proteins [62,66].

The major function of SPRY proteins is the interference with the RAS/ERK pathway [61,67]. SPRY2 interacts with GRB2 and RAF (Figure 3), thereby interfering with the ERK pathway upstream and downstream of RAS [58,67,68]. Among the different SPRY isoforms, SPRY2 reveals the strongest inhibitory effect on ERK activation [67,69]. In addition to the ERK pathway, SPRY2 inhibits the activation of phospholipase C (PLC), RAS-related C3 botulinum toxin substrate 1 (RAC1) and Akt in some reports [70,71,72,73]. In adult DRG neurons the downregulation of SPRY2 leads to the activation of ERK in response to FGF2 treatment, whereas phosphorylation of Akt remains unchanged [21,74].

SPRY proteins are regulated at the transcriptional and the post-translational level, and the expression of SPRY is influenced by growth factors through ERK activation [21,63,75,76]. All SPRY proteins contain a highly conserved C-terminal cystein-rich region and a variable N-terminal region with various phosphorylation sites (Figure 2). Several kinases, phosphatases and ubiquitin ligases interact with SPRY [77]. Kinases such as dual-specificity tyrosine-phosphorylated and -regulated kinase 1A (DYRK1A), testicular protein kinase 1 (TESK1) or MAPK-interacting kinase 1 (Mnk1) and phosphatases such as PTEN, protein phosphatase 2A (PP2A), Src homology 2-containing phosphotyrosine phosphatase (SHP2) or protein tyrosine phosphatase 1B (PTP1B) regulate the biological activity of SPRY [78]. Dephosphorylation of SPRY2 by PP2A results in a conformational change at the C-terminal proline-rich binding site for GRB2, thereby preventing the interaction of GRB2 with SOS and subsequent ERK activation, while its dephosphorylation by SHP2 dissociates it from GRB2 [68,79,80]. The E3 ubiquitin ligases c-casitas b-lineage lymphoma (c-CBL) and seven in absentia homolog 2 (SIAH2) interact with the N-terminus and control ubiquitination and degradation of SPRY2 [81,82]. Furthermore, miR-21 downregulates SPRY2 at the post-transcriptional level [83,84].

### 3.1. SPRY and Development 

SPRY proteins were first studied in the NS during development. In ovo electroporation of dominant-negative SPRY2 results in an anterior shift of the posterior border of the tectum during brain development, whereas overexpression of SPRY2 induces a fate change in the presumptive metencephalon to the mesencephalon [85]. SPRY1 and SPRY2 are expressed at higher levels in the developing cerebellum than SPRY4 [86]. In primary cultures of immature cerebellar granule neurons, inhibition of SPRY2 promotes neurite outgrowth [63]. Cerebellar development is only mildly affected in SPRY2 knockout mice, but the additional deletion of other SPRY isoforms results in severe developmental defects of the cerebellum [86]. SPRY2 is expressed higher in the developing hippocampus than SPRY4, and both SPRY isoforms are reduced up to 14 days postnatally compared to embryonic day 16.5. In embryonic hippocampal neuron cultures, SPRY2 and SPRY4 are concentrated in growth cones of axons and dendrites, and knockdown of SPRY2 or SPRY4 enhances axon outgrowth by hippocampal neurons which is further increased by treatment with FGF2 [76]. These data from developing neurons indicate a role of SPRY in axon regeneration. 

**Figure 4:**
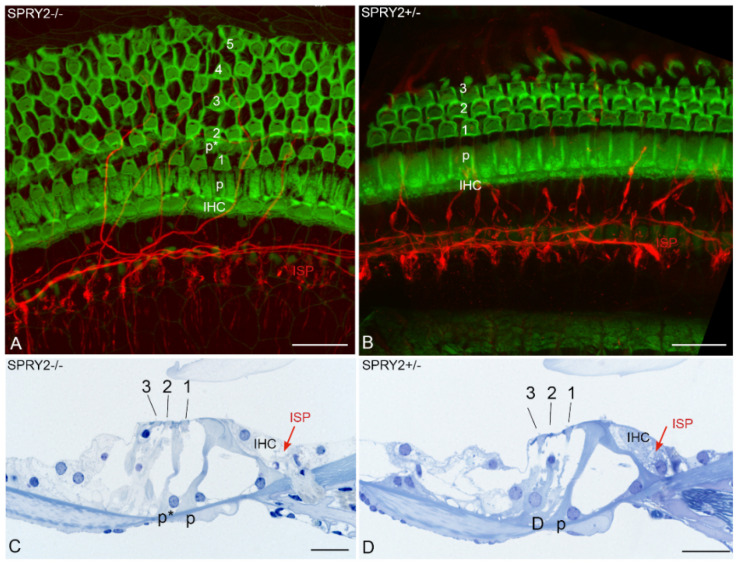
Middle turns of the cochlea from one-month-old SPRY2−/− (**A**,**C**) and SPRY2+/− (**B**,**D**) knockout mice. Whole mount preparations (**A**,**B**) were stained with fluorescent phalloidin (green) which labels the actin-rich hair cells and an anti-neurofilament 200 antibody (red) labeling nerve fibers. Images were acquired with a confocal microscope. SPRY2−/− mice revealed up to five rows of outer hair cells (OHCs; **A**, 1–5) that are arranged less ordered compared to SPRY2+/− mice that reveal the typical regular mosaic pattern of sensory and supporting cells with three rows of OHCs (**B**, 1–3). Afferent and efferent nerve fibers intermingle in the inner spiral plexus (ISP) underneath the inner hair cell (IHC) and appear normal. The additional pillar cell (p*) is best visible in plastic embedded semithin sections (**C**,**D**). D shows the conventional cytoarchitecture in a SPRY2+/− littermate with a normal outer pillar cell (p). An additional outer pillar cell (p* in subfigure (**C**)) resembles more the first row of Deiters’ cells (D in subfigure (**D**)). Scale bars = 20 µm.

In addition to brain development, SPRY proteins are also involved in the development of the enteric NS. SPRY2 knockout mice reveal enteric nerve hyperplasia induced by GDNF-induced hyperactivation of ERK that leads to esophageal achalasia [64]. In the inner ear, homozygous SPRY2 deficiency disrupts the delicate cytoarchitecture of the organ of Corti. Additional pillar cells due to a cell fate transformation of a Deiters’ cell into a pillar cell in the early postnatal period and augmentation of the motile cochlear amplifying outer hair cells (OHCs) are observed in response to SPRY2 knockdown [87]. The pillar cells form the inflexible triangulated tunnel of Corti that changes in dimensions and stiffness from high to low auditory frequencies [88]. This unique architecture together with the active mechanical feedback of OHCs deforms the organ of Corti in a complex way that is indispensable for the extraordinary sensitivity, dynamic range and tuning of hearing. Our recent results confirmed that SPRY2 knockout mice reveal changes in the cytoarchitecture of the organ of Corti. Homozygous SPRY2−/− mice reveal four to five rows of OHCs with less regular planar cell polarity in contrast to the conventionally ordered three rows of OHCs in heterozygous SPRY2+/− mice (Figure 4). Whether these cytoarchitectural changes are the only abnormalities or other yet hidden changes contribute to the hearing loss of 60 decibels in SPRY2−/− knockout mice and 7 decibels loss between SPRY2−/− and SPRY2+/− [87] is unknown. However, auditory neurons (data not shown) and sensory epithelium innervations (Figure 4) appeared normal indicating that the absence of SPRY2 does not affect fiber guidance. FGF8 induces pillar cell fate and regulates cellular patterning in the cochlea [89]. While the conditional deletion of FGF8 reduces the number of pillar cells, SPRY2 deletion induces additional pillar cells (Figure 4). This effect in SPRY2−/− knockout mice is partially rescued by reducing the FGF8 dosage, indicating that SPRY2 prevents the cell fate transformation of a Deiters’ cell into a pillar cell by the inhibition of FGF8 signaling [87].

### 3.2. SPRY and Axonal Regeneration 

Inhibition of SPRY2 reduces apoptotic cell death in primary cultures of mature cerebellar granule neurons [63]. In addition to its effects on axon growth during the development of cerebellar and hippocampal neurons, deletion of SPRY2 promotes neurite outgrowth by adult DRG neurons [21,74]. Although SPRY2 mRNA is not altered in response to a sciatic nerve lesion, SPRY2 is regulated post-transcriptionally in the DRG by miR-21 in response to a peripheral nerve transection. Furthermore, the overexpression of miR-21 promotes neurite outgrowth by adult DRG neuron cultures on a reduced laminin substrate [21,90]. MiR-21 is upregulated up to 28 days after axotomy and heterozygous SPRY2+/− knockout mice recover faster in response to a sciatic nerve crush revealing a larger diameter of the distal sciatic nerve, higher numbers of myelinated axons and a higher density of motor endplates. Homozygous SPRY2−/− mice reveal enhanced mechanosensory function (demonstrated by the von Frey test) that is accompanied by increased innervation of the epidermis and elevated numbers of non-myelinated axons [74,90]. 

In addition to PNS regeneration, the inhibition of SPRY proteins enhances the regeneration of the damaged brain as well. Simultaneous partial knockdown of SPRY2 and SPRY4 limits secondary brain damage in response to kainate-induced epileptogenesis. Neurodegeneration and granule cell dispersion are alleviated following kainic acid-induced hippocampal lesion in SPRY2/4 double heterozygous knockout mice [91]. Injection of SPRY2/4 siRNAs into the rodent brain diminishes the size of the lesion after endothelin-induced vasoconstriction, a model for human stroke [92]. Taken together, SPRY is a promising target to enhance regeneration in the PNS and the CNS (Figure 3).

## 4. PTEN: Signal Regulator of the PI3K/Akt Pathway

PTEN was originally identified in 1997 as a tumor suppressor gene that is mutated in several tumors, including glioblastoma [93,94]. Starting from one report in 2008 demonstrating that the deletion of PTEN promotes robust axonal regeneration in the CNS [95], PTEN became of emerging interest in the field of neuronal regeneration. PTEN is a dual phosphatase that can act on both phosphoinositide and polypeptide substrates (Figure 2). Through its lipid phosphatase activity, PTEN converts PIP3 to PIP2 (Figure 3) by dephosphorylating the 3’ position of the inositol ring in PIP3, thereby reversing the reaction catalyzed by PI3K [96]. As a consequence, downstream signaling of PI3K including pAkt and mTOR is inhibited [5]. PTEN inhibits PI3K signaling in response to a plethora of growth factors including NGF [97], BDNF [98], GDNF [99], FGF [100,101], EGF [102] and CNTF [103]. 

As an important inhibitor of PI3K signaling, the expression of PTEN is regulated at multiple levels. Numerous miRs modulate PTEN expression at the post-transcriptional level. Among them miR-21, miR-222, miR-26a and miR-182 suppress PTEN expression in DRG or cortical neurons [104,105,106,107,108,109]. PTEN is downregulated by several E3 ubiquitin ligases including neural precursor cell expressed developmentally down-regulated protein 4 (NEDD4) or X-linked inhibitor of apoptosis protein (XIAP) which are both involved in axonal regeneration [110,111,112]. PTEN is co-expressed with its ubiquitin ligase NEDD4 in DRG neurons, in the sciatic nerve and in growth cones of RGCs [111,113]. 

Phosphorylation of the C-terminus of PTEN (Figure 2) inhibits its lipid phosphatase activity and enhances its stability [114]. PTEN phosphorylation mediated by GSK3 induces the interaction of PTEN with myosin V. Abolishment of the interaction between PTEN and myosin V increases the neuronal soma size similar to PTEN inhibition [115,116]. The translocation of PTEN into the nucleus is mediated through mono-ubiquitination by NEDD4 and different other mechanisms. The lipid phosphatase activity of PTEN, which interferes with pAkt signaling, predominates in the cytoplasmic compartment, whereas the protein phosphatase activity is generally nuclear [117,118]. PTEN levels are enhanced in small isolectin B4 (IB4)-positive neurons which are limited in their regenerative capability [119,120]. Glial cells in DRG cultures exhibit reduced PTEN levels [53]. Phosphorylated PTEN is highly expressed in the nuclei of large and medium-sized DRG neurons [120]. In the adult brain, PTEN is preferentially expressed in neurons especially in Purkinje neurons, olfactory mitral neurons and large pyramidal neurons but not in astrocytes or oligodendrocytes [121]. Thus, PTEN expression appears to be mainly neuronal in the PNS and in the CNS.

### 4.1. PTEN and Development 

In the rodent brain, PTEN protein levels increase starting at E17 and remain stable during postnatal development and adulthood [121,122]. During development, axonal PTEN activity is inhibited by plasticity-related gene 2 (PRG2), a protein which targets PTEN at axon branch points and stabilizes membrane PIP3. PRG2 protein levels in the rodent brain increase from E17 to P1 corresponding to cortical migration and branch formation of axon projections [122]. Furthermore, miR26a targeting PTEN is upregulated in the hippocampus after birth and promotes axon growth by hippocampal neurons [109]. PTEN deletion in the brain during early development reveals neuronal overgrowth, brain enlargement, seizures and premature death. Depending on the promotor used in the different mouse models of PTEN deletion, mice exhibit higher postnatal mortality or premature death in the first weeks of life [123]. Deletion of PTEN enhances axon outgrowth by developing hippocampal neurons through an increase in detyrosinated, stable microtubules [124].

PTEN is transiently expressed during cochlea development in hair cells (HCs) and it is downregulated at postnatal day seven [125]. The number of HC progenitors that differentiate into HCs is augmented in heterozygous PTEN+/− knockout mice and after conditional PTEN knockout in the inner ear by increased activation of Akt. The levels of the downstream target cyclin-dependent kinase inhibitor p27^kip1^ decrease which prevents HC progenitors from cell cycle exit. Furthermore, the cytoskeletal differentiation of HCs is affected by PTEN deletion [126,127]. Differentiation is a critical step during HC development and this process is to some extent regulated by FGF20. Inhibition or deletion of FGF20 reduces the HC number and this effect is partially rescued by PTEN inhibition [128,129,130]. PTEN is present in the cytoplasm of embryonic DRG neurons and at low levels in the nucleus, while it is highly enriched in the axonal compartment during axon extension. PTEN protein accumulates in the central domain of the growth cone of embryonic DRG neurons where it associates with microtubules [131]. PTEN increases in the DRG and in the sciatic nerve postnatally in neuronal cell bodies and axons. Apparently, the expression of PTEN in peripheral nerves is reduced preceding myelination [132]. 

### 4.2. PTEN and Axonal Regeneration

The first study about PTEN in axonal regeneration revealed that PTEN deletion enables CNS regeneration after crush injury in the adult optic nerve through the activation of Akt and subsequent downstream signaling of mTOR and phosphorylation of S6. In addition, deletion of PTEN reduces retrograde degeneration of RGCs demonstrating a positive effect on neuron survival [95]. The E3 ubiquitin ligase NEDD4 is expressed in RGC axonal growth cones together with PTEN and the disruption of NEDD4 inhibits terminal branching through PTEN [113]. Studies about the effects of PTEN deletion in corticospinal tract (CST) regeneration confirmed the high potential of PTEN to enhance CNS regeneration. Conditional deletion of PTEN before or following spinal cord injury promotes axon regeneration across the lesion site, which is followed by the enhanced recovery of motor functions [133,134,135,136]. Remarkably, PTEN deletion even induced CST regeneration in a chronic injury model one year after injury [137]. Since PTEN deletion is common in several cancers and disrupts brain development leading to neurological abnormalities including brain enlargement, seizures and early mortality, deletion of PTEN in the CNS may lead to adverse side effects. An important study demonstrated that the deletion of PTEN one day after birth in the motor cortex of mice did not cause evident pathology such as tumors after 12–18 months. However, cortical motoneurons lacking PTEN were larger and the laminar organization of the cortex in the area of the PTEN deletion was disrupted [138]. 

Studies in the PNS confirmed the effects of PTEN inhibition on axonal regeneration. Sciatic nerve injury reduces PTEN mRNA and protein levels gradually post injury in the DRG and the sciatic nerve [104,120,132]. PTEN protein is reduced as well in DRG neuron cultures after 72 h during rapid axon outgrowth on laminin substrate [53]. Pharmacological inhibition or deletion of PTEN promotes axon outgrowth of adult DRG neurons in vitro. In contrast to the CNS, neurite outgrowth of DRG neurons in response to PTEN inhibition is independent of the mTOR pathway, but it requires the activity of PI3K and Akt [53,95,120]. Knockdown of the PTEN-regulating ubiquitin ligase NEDD4 upregulates PTEN in DRG neurons and impairs axon outgrowth [111]. MiR-222 is upregulated in DRG neurons after sciatic nerve transection which suppresses PTEN and promotes neurite outgrowth in vitro [104]. Pharmacological inhibition and deletion of PTEN promotes regeneration and enhances myelination of the sciatic nerve after transection or crush injury [120,132]. A conditioning injury of the sciatic nerve leads to the release of NADPH oxidases from inflammatory macrophages in exosomes at the lesion site, which are retrogradely transported to the cell body and decrease the PTEN activity by oxidation, thereby increasing Akt activation [139]. PTEN mRNA and protein are upregulated in diabetic mice, indicating the association of PTEN with diabetic regenerative failure. Thus, PTEN knockdown also accelerates the recovery after sciatic nerve injury in a chronic model of diabetic polyneuropathy [140]. These various studies in the PNS underscore the multiple functions of PTEN inhibition to promote axonal regeneration (Figure 3). 

## 5. Combined Approaches to Enhance Axonal Regeneration

Regeneration of the CNS is still highly limited despite major efforts being made in research. In the PNS regeneration is possible; however, the functional outcomes are often poor in patients [3,15,141]. Neuronal regeneration is influenced by multiple factors, and recent studies concurrently targeting different regulators of axon growth and signaling pathways are promising. Our recent study demonstrated that simultaneous deletion of SPRY2 and PTEN promotes axon elongation by the activation of Akt in adult DRG neuron cultures stronger than the single knockdown. Interestingly, PTEN protein was significantly reduced in DRG cultures from homozygous SPRY2 knockout mice, and PTEN deletion strongly reduced SPRY2 protein levels indicating the reciprocal regulation of SPRY2 and PTEN in DRG neurons [53]. Bisperoxovanadium (bpV) compounds such as bpV(pic) or bpV(phen) serve as PTEN inhibitors [98,103,120,142]. To the best of our knowledge inhibitors for SPRY2 are not available to date (SPRY proteins have no enzymatic function). MiRs targeting SPRY2 and PTEN may be useful for therapeutic approaches. MiR-21 reduces SPRY2 in the DRG and promotes neurite outgrowth by adult DRG neuron cultures [90]. In cortical neurons, miR-21 decreases the expression of PTEN and reduces neuronal apoptosis after scratch injury in vitro [106]. Furthermore, numerous miRs inhibit PTEN in tumors and other disorders such as stroke. A recent review provides a comprehensive overview about miRs that regulate PTEN [143]. In addition to miR-21, SPRY2 is regulated by the miR-23a/24-2/27a cluster and by miR-122, miR-124 or miR-330-5p [144,145,146,147]. Among these miRs in tumors, miR-23a inhibits SPRY2 and PTEN. Thus, miRs targeting different inhibitors may be useful for future therapies. In vivo studies in the PNS investigated the effect of the conditional co-deletion of PTEN and the suppressor of cytokine signaling 3 (SOCS3), an inhibitor of the Janus kinase (JAK)/signal transducer and activator of transcription (STAT) pathway using PTEN/SOCS3^fl/fl^ floxed mice. Although co-deletion of PTEN and SOCS3 increases sciatic nerve regrowth after crush lesion to a similar extent as PTEN deletion alone, simultaneous knockdown of PTEN and SOCS3 leads to more rapid recovery of noxious thermo- and mechanosensation than PTEN deletion alone [148].

In the CNS, simultaneous knockdown of PTEN and SOCS3 reveals improved regeneration after optic nerve crush lesion [149]. Furthermore, genetic deletion of SOCS3 significantly promotes optic nerve regeneration when combined with intravitreous injection of CNTF [150]. Likewise, PTEN knockdown using adeno-associated virus (AAV) vectors combined with AAV encoding CNTF or cyclic adenosine monophosphate (cAMP) analogue, enhances RGC axon regeneration stronger than PTEN downregulation alone [151]. The combination of PTEN deletion with the elevation of intraretinal cAMP activity by applying a cAMP analogue and additional induction of eye inflammation by Zymosan injection, enables RGCs to regenerate axons the full length of the visual pathway. This strong stimulation of optic nerve regeneration even partially restores optomotor response, depth perception and circadian photoentrainment, demonstrating that combined approaches are utterly necessary for functional regeneration to occur [152]. In addition to optic nerve regeneration, CST regeneration induced by the transduction of cortical motoneurons with hyper-interleukin-6 (hIL-6) to stimulate JAK/STAT3 signaling, is further enhanced by PTEN deletion [153]. Thus, the simultaneous stimulation of different signaling pathways that promote neuronal regeneration is promising for the development of novel treatments.

## 6. Conclusions

Together these studies demonstrate the outstanding role of signal transduction regulators in axonal regeneration of the PNS and CNS. In particular, the deletion of the signal transduction regulators SPRY2 and PTEN improves axonal regeneration in the CNS in ways that have scarcely been observed before. Approaches targeting both SPRY2 and PTEN or combining PTEN deletion with cAMP, CNTF or hIL-6 had even greater effects, holding great promise for new treatments of neurological injuries in the future.
